# Novel Note Templates to Enhance Signal and Reduce Noise in Medical Documentation: Prospective Improvement Study

**DOI:** 10.2196/41223

**Published:** 2023-04-12

**Authors:** Jonah Feldman, Adam Goodman, Katherine Hochman, Eesha Chakravartty, Jonathan Austrian, Eduardo Iturrate, Brian Bosworth, Archana Saxena, Marwa Moussa, Dina Chenouda, Frank Volpicelli, Nicole Adler, Joseph Weisstuch, Paul Testa

**Affiliations:** 1 Medical Center Information Technology NYU Langone Health New York, NY United States; 2 Department of Medicine NYU Long Island School of Medicine Mineola, NY United States; 3 Division of Gastroenterology & Hepatology NYU Grossman School of Medicine New York, NY United States; 4 Department of Medicine NYU Langone Health New York, NY United States; 5 NYU Langone Health Brooklyn, NY United States

**Keywords:** medical informatics, decision support, hospital data, clinical documentation, clinical informatics

## Abstract

**Background:**

The introduction of electronic workflows has allowed for the flow of raw uncontextualized clinical data into medical documentation. As a result, many electronic notes have become replete of “noise” and deplete clinically significant “signals.” There is an urgent need to develop and implement innovative approaches in electronic clinical documentation that improve note quality and reduce unnecessary bloating.

**Objective:**

This study aims to describe the development and impact of a novel set of templates designed to change the flow of information in medical documentation.

**Methods:**

This is a multihospital nonrandomized prospective improvement study conducted on the inpatient general internal medicine service across 3 hospital campuses at the New York University Langone Health System. A group of physician leaders representing each campus met biweekly for 6 months. The output of these meetings included (1) a conceptualization of the note bloat problem as a dysfunction in information flow, (2) a set of guiding principles for organizational documentation improvement, (3) the design and build of novel electronic templates that reduced the flow of extraneous information into provider notes by providing link outs to best practice data visualizations, and (4) a documentation improvement curriculum for inpatient medicine providers. Prior to go-live, pragmatic usability testing was performed with the new progress note template, and the overall user experience was measured using the System Usability Scale (SUS). Primary outcome measures after go-live include template utilization rate and note length in characters.

**Results:**

In usability testing among 22 medicine providers, the new progress note template averaged a usability score of 90.6 out of 100 on the SUS. A total of 77% (17/22) of providers strongly agreed that the new template was easy to use, and 64% (14/22) strongly agreed that they would like to use the template frequently. In the 3 months after template implementation, general internal medicine providers wrote 67% (51,431/76,647) of all inpatient notes with the new templates. During this period, the organization saw a 46% (2768/6191), 47% (3505/7819), and 32% (3427/11,226) reduction in note length for general medicine progress notes, consults, and history and physical notes, respectively, when compared to a baseline measurement period prior to interventions.

**Conclusions:**

A bundled intervention that included the deployment of novel templates for inpatient general medicine providers significantly reduced average note length on the clinical service. Templates designed to reduce the flow of extraneous information into provider notes performed well during usability testing, and these templates were rapidly adopted across all hospital campuses. Further research is needed to assess the impact of novel templates on note quality, provider efficiency, and patient outcomes.

## Introduction

### Background and Significance

Since the Health Information Technology for Economic and Clinical Health (HITECH) Act was enacted, provider notes have lengthened with an increase in note redundancy [1]. Across the same period, US notes have been reported as 4 times longer than those in other countries [2]. Some claim that poor note quality is one of the unintended consequences of nationwide electronic health record (EHR) adoption [3-6]. The introduction of electronic workflows has allowed the flow of raw uncontextualized clinical data into provider notes [5,7-9]. Over time, and with the change in the culture of clinical practice, many electronic notes have become full of noise, and clinically significant signals are now  becoming harder to find [1,3,5,9,10]. There is an urgent need to develop and implement innovative approaches in electronic clinical documentation that improve note quality and reduce unnecessary bloating [5,8,9].

### Sequence and Flow in the EHR

Significant unintended consequences to care associated with the adoption of EHR are rooted in unplanned changes to the sequence of complex clinical events. EHR interventions can have unintended changes to workflow, which is defined as the sequence of activities necessary to complete a task, and patient flow, which is described as the ordered movement of patients in a health care setting. Examples of disordered workflow after EHR adoption include changes to sequence of complex physician ordering processes after the implementation of computerized physician order entry [11] and changes to temporal trends in physician task workflow as well as increased time spent in clinical review and documentation [12]. Some examples of changes in patient flow included a lack of understanding of the sequence of patient event after transition from paper to electronic flowsheets [13] and disordered patient flow with the requirement that staff need physician orders prior to initiating “routine” clinical testing [14].

### Sequence and Flow in Clinical Thinking and Communication

Sequence also plays a role in clinical thinking and communication processes. SBAR (situation, background, assessment, and recommendation) [15], IPASS (illness severity, patient summary, action list, situation awareness, and contingency planning and synthesis by receiver) [16], and SOAP (subjective, objective, assessment, and plan) [17] are frameworks, or mental models, that advance ordered standards for thought and communication. SBAR and IPASS are used at the time of clinical handoff, and SOAP is a construct for clinical notes. These standards promote a sequential structure where adherence to sequence is a key element to improve patient safety and impact other clinical outcomes [18-20]. In this context, the Data, Information Knowledge, Wisdom (DIKW) framework is a valuable tool that has been used to study clinical communication [21-24]. The framework is composed of 4 levels, each building on the prior. Data represent the initial stage, where raw facts such as patient vital signs and laboratory results are collected. Information is the next stage, where data such as vitals trend are organized and processed. Knowledge is the third stage, where information such as diagnosis or assessment is interpreted and applied. Wisdom is the final stage, where knowledge such as considering a patient’s cultural and personal values when making a treatment plan is applied in a practical and integrated manner. By using the DIKW framework, researchers can analyze the flow of communication and identify any gaps or inefficiencies in the sequential progression from data to wisdom [25-30].

### Sequence and Flow in Clinical Notes

**Figure 1:**
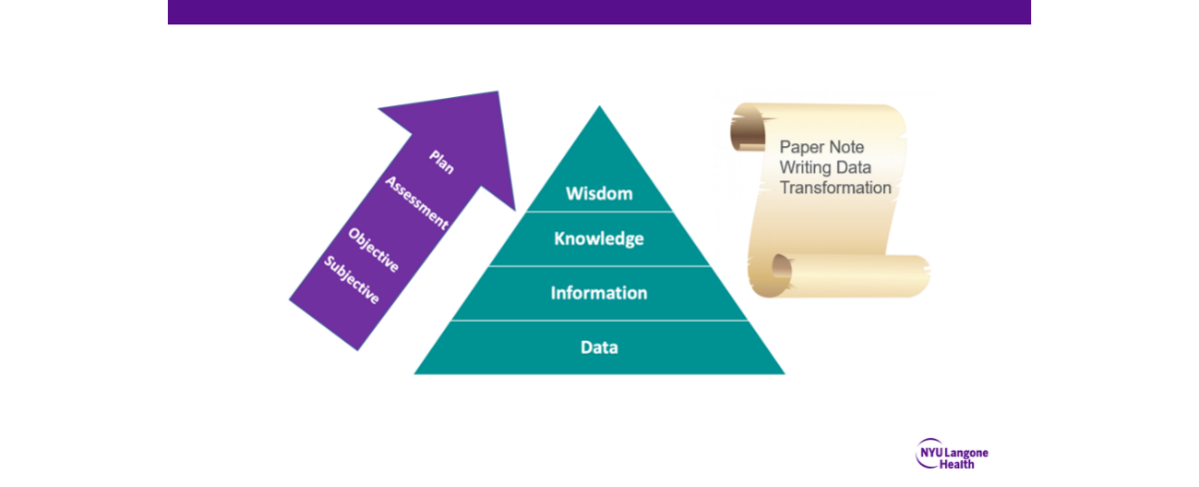
Data Information Knowledge Wisdom pyramid.

Considering the importance of sequence and flow to clinical thinking, clinical communication, and EHR workflows in general, it is not surprising that electronic note-writing introduced features that change the flow of information and can impact the output of the note composition. The original SOAP note structure, created by Larry Weed, was intended as a data transformation engine that takes raw clinical data and transforms it into information in the “Subjective” and “Objective” sections [31]. For instance, the patient may report a headache and difficulty sleeping (subjective), and the clinician may find that the patient’s vital signs are within normal limits but with tenderness in the temples upon physical examination (Objective). This information is then converted into knowledge in the “Assessment” section, such as the conclusion that the patient is likely experiencing a headache due to stress and lack of sleep. Finally, the knowledge is transformed into wisdom in the “Plan” section, with a recommendation for over-the-counter pain relief medication and advice for the patient to practice stress management techniques and establish a consistent sleep schedule. In this way, the note writer sequentially [32,33] ascends the DIKW pyramid [21], while the SOAP note is composed (Figure 1). Modern electronic note-writing workflows have disrupted this time-tested process for data transformation by allowing data and information to flow directly from the EHR into predefined note sections before the clinician thinks about the patient’s condition. Untimely information flow into note templates can thus be viewed as a root cause of poor note quality and a major contributor to the note bloat epidemic.

### Study Objective

**Figure 2:**
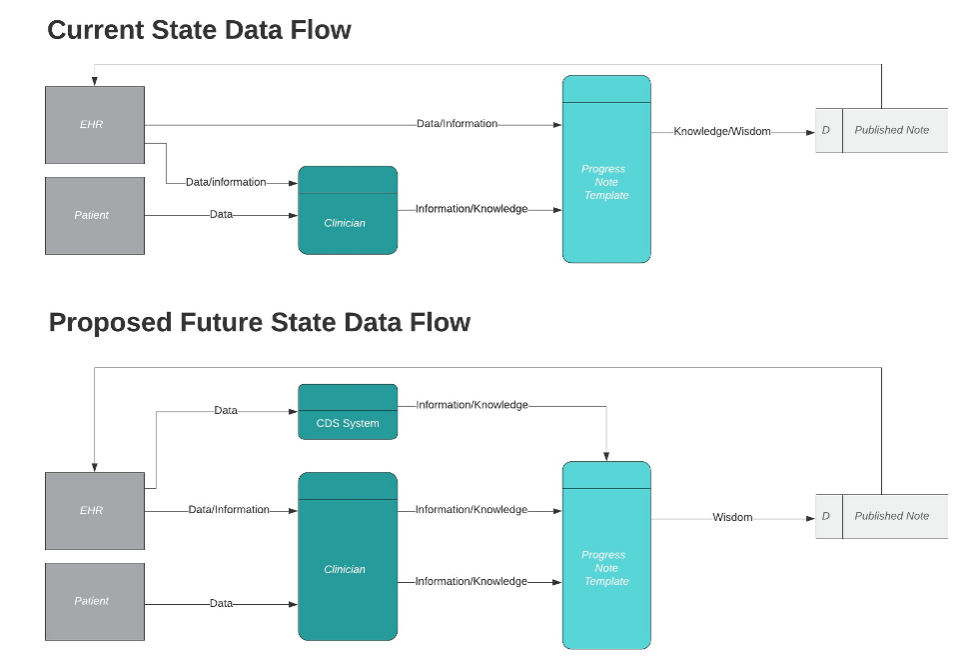
Current state data flow: unprocessed data flows directly from the EHR into provider notes, proposed future state data flow: only data processed into information/knowledge by a clinician or CDS system flows into provider notes. CDS: clinical decision support; EHR: electronic health record.

The objective of this study is to describe the development and impact of a novel set of templates designed to change the flow of information into provider notes (Figure 2).

We describe the development process, including a description of institutional governance, guiding principles that informed template design, template creation, usability testing, and implementation.

### Pragmatic Usability Testing

Usability testing is essential to the EHR implementation life cycle [34-38], and testing events can help promote provider adoption [39,40]. Combining “near-live” clinical simulation with “think-aloud” protocols has been shown to help assess user preference and impact on workflow [41]. “Think-aloud” protocols require users to verbalize their thought process while interacting with a new clinical tool [41-44]. “Near-live” testing allows for a more fluid environment to identify further real-life barriers [34,41]. “Near-live” clinical simulation with “think-aloud” protocols can be deployed together with quantitative usability assessment like the System Usability Scale (SUS) [45-47] as part of a pragmatic usability test strategy [34] that prioritizes speed and cost-effectiveness. The SUS has been used extensively to assess the usability of the EHR [42,48-50], including use to assess electronic note template usability [51]. The 10 statements on the SUS use a 5-point Likert scale to measure the strength of agreement or disagreement with each statement. The total composite score is a number from 0 to 100, with high SUS scores indicating greater usability and satisfaction.

## Methods

### Institutional Setting

This is a multihospital nonrandomized prospective improvement study conducted on the inpatient general internal medicine (GIM) service across 3 hospital campuses at the New York University Langone Health System (NYULH). NYULH is a large academic health care system in New York, consisting of over 5000 health care providers across multiple hospitals and >500 ambulatory locations. NYULH’s implementation of a single EHR (Epic Systems Corporation) instance and seamless integration of ancillary systems help to facilitate a single clinical standard throughout the enterprise. To support measurement and continuous improvement for this project, EHR data were queried from the Epic System’s Clarity database using SQL Developer (Oracle Corporation) and exported for analysis of note length and template adoption. The note templates were implemented in the November 2020 version of Epic. Prior to our quality improvement intervention, the institution’s instance of Epic does not allow “copy forward” in the inpatient setting. NYULH did not have any standardized note templates prior to our intervention.

### Ethical Considerations

We followed the NYU Grossman School of Medicine institutional review board (IRB) protocol and completed an IRB checklist for activities that may be classified as quality improvement. This work, including our usability study, met the IRB criteria for quality improvement and therefore did not require IRB review or informed consent.

### Documentation Improvement Bundle and Template Development

**Figure 3:**
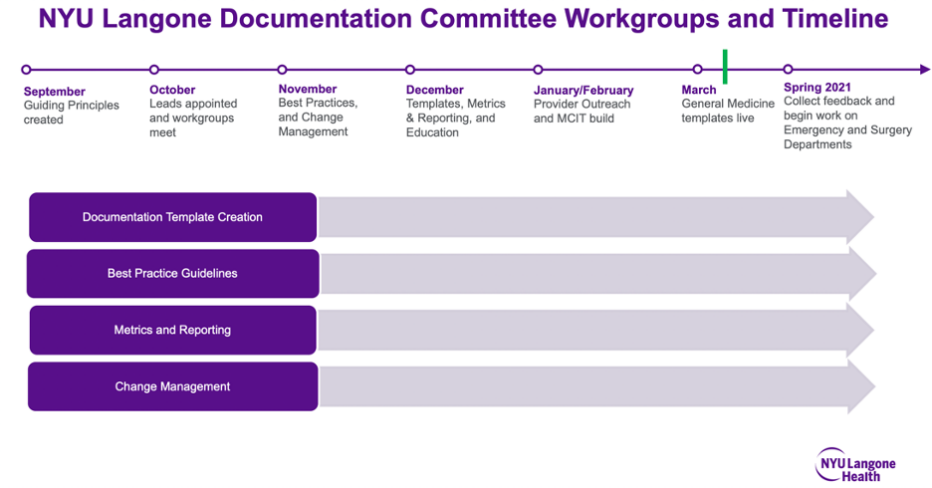
Timeline and committee structure for an improvement initiative. The green line represents the implementation of GIM standard note templates. GIM: general internal medicine; MCIT: Medical Center Information Technology; NYU: New York University.

**Figure 4:**
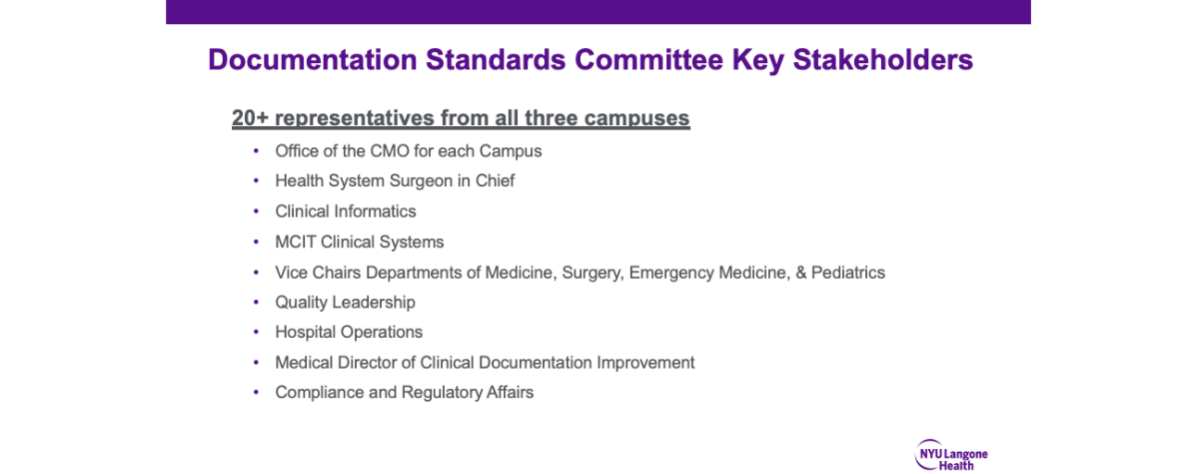
Key committee stakeholders. CMO: Chief Medical Officer; MCIT: Medical Center Information Technology.

Our documentation improvement efforts began in September 2020, when a group of physician leaders from NYULH representing each hospital campus began to meet biweekly as part of the newly formed Documentation Standards Committee. The committee’s stated goal was to develop and implement standardized documentation and accountability processes for provider notes to improve quality and readability, while eliminating errors, and capturing the complexity of care provided. Figure 3 represents the timeline for the improvement initiative and committee structure, respectively. Figure 4 is a list of key committee stakeholders.

**Figure 5:**
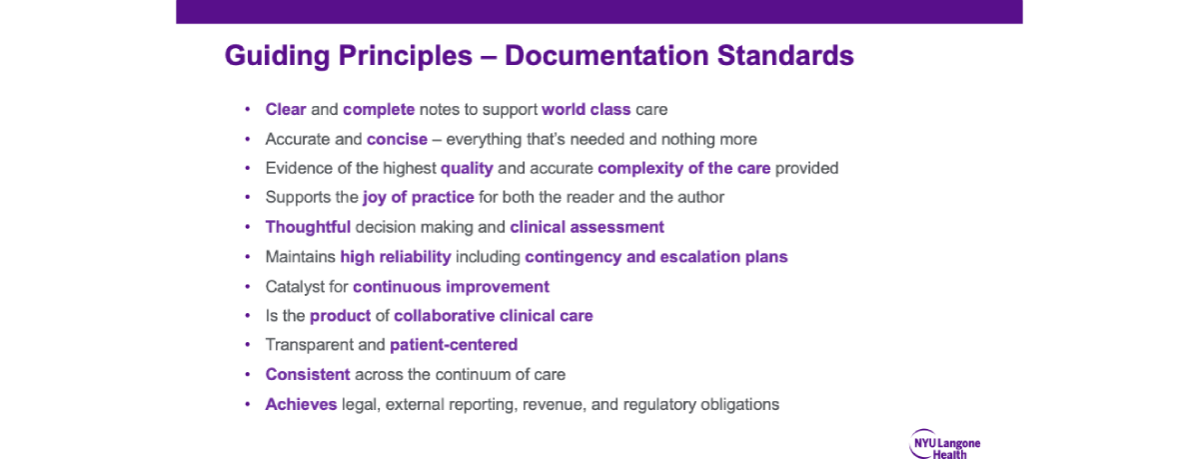
Guiding principles.

In the first 6 months, during the committee meetings, we conceptualized note bloat. As a result of these committee meetings, we introduced the bundled intervention that included (1) conceptualization of the note bloat problem as a dysfunction in information flow (Figures 1 and 2); (2) a set of guiding principles for organizational documentation improvement (Figure 5); (3) a documentation improvement curriculum for inpatient medicine providers; and (4) the design and build of novel new electronic templates that sought to reduce the flow of extraneous information into provider notes by providing link outs to best practice data visualizations.

### Description of Template Features and Functions

**Figure 6:**
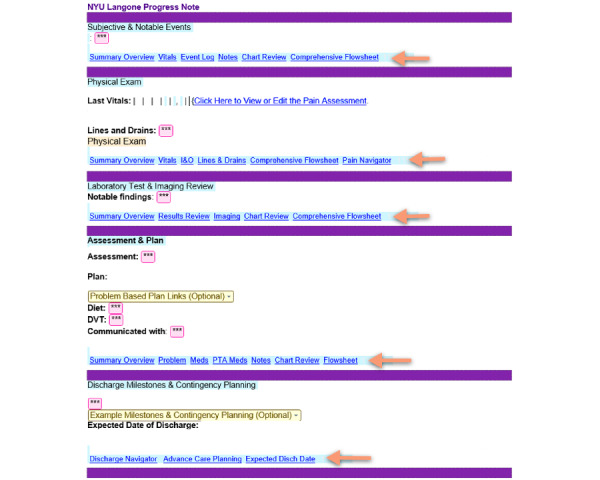
Progress note template for general internal medicine with hyperlinks (arrows). DVT: deep vein thrombosis; I&O: input and output; NYU: New York University; PTA: prior to admission.

Figure 6 shows the progress note template developed for the inpatient GIM service. The first implementation of new standard templates was for GIM and included: progress notes, consults, and history and physical (H&P) notes. Within these new templates, very little clinical data flow directly into the note. Instead, each template section has hyperlinks that link out to best visualizations that appear next to the template to support clinical data review. These hyperlinks provide direct access to data-specific sections within the patients’ medical record including medical and surgical history; for example, the data are visualized in a side panel next to the open note. This allows for clinical data review and simultaneous entry of relevant details into the clinical note. Providers are prompted within the template to document a synthesis of the information or knowledge relevant to the note section. Each note section contains a unique combination of hyperlinks with tailored data visualizations to support appropriate clinical thinking in that part of the note. All the hyperlinks disappear at the time of note publication.

The templates generally follow the SOAP note structure, although additional sections were added for clarity or to adapt to the modern practice of medicine. The template sections are titled as follows: Subjective & Notable Events, Physical Exam, Laboratory Test & Imaging Review, Assessment & Plan, and Discharge Milestones & Contingency Planning. This last section continues the sequential progression of the SOAP note structure by moving onto future and contingency planning, including how the patient's care is affected by health system considerations about the advancement of care and hospital discharge.

**Figure 7:**
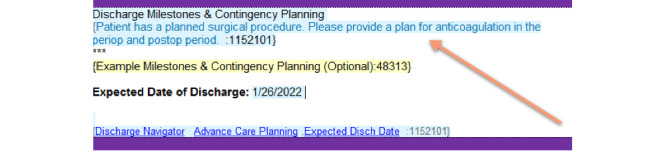
Disappearing tip (arrow) for anticoagulation in patients with a planned surgical procedure on full-dose anticoagulation for up to 48 hours prior to surgery.

**Figure 8:**
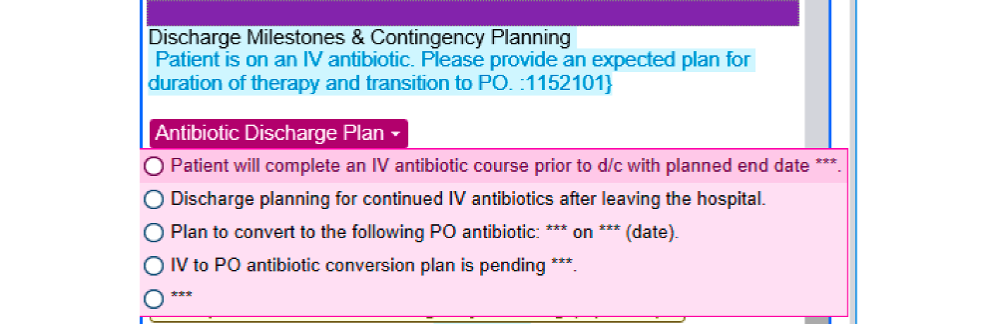
Disappearing tip for antibiotic plan in patients on intravenous antibiotics and expected time of discharge in the next 24 hours. d/c: discharge; IV: intravenous; PO: by mouth.

An additional feature of these novel templates is the presence of patient-specific CDS presenting as disappearing tips that appear in the body of the note. Figure 7 demonstrates a CDS nudge directed at providers to consider the plan for anticoagulation in patients who are on full-dose anticoagulation up to 48 hours prior to surgery. Figure 8 is an example of another CDS nudge to providers to consider the plan for antibiotics, upon discharge, for patients who are currently on intravenous antibiotics. The usability of CDS nudges within note sections was evaluated as part of formal usability testing as described below.

### Usability Participation, Procedure, and Analysis

Prior to go-live, pragmatic usability testing was performed with the new templates. Four board-certified physician informaticists served as facilitators for 45-minute one-on-one testing sessions with frontline providers. A purposive sample of inpatient internal medicine providers was selected: 11 from NYU Langone—Long Island Hospital, 3 from NYU Langone—Brooklyn Hospital, and 8 from the Tisch and Kimmel Hospitals on the Manhattan campus. Half of the providers were attending physicians and half were residents or physician assistants. All the providers routinely write notes on hospitalized general medicine service patients.

**Figure 9:**
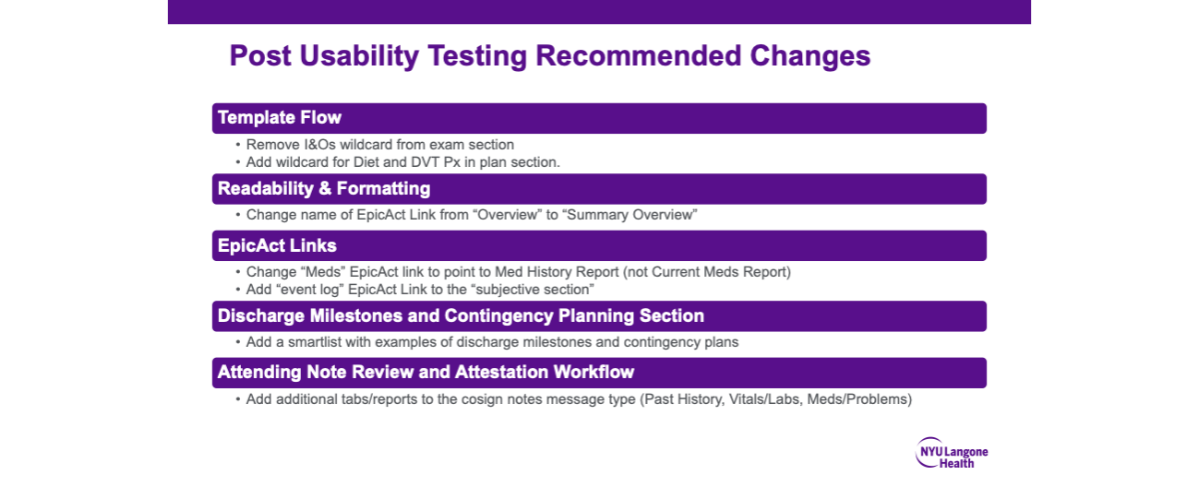
Improvements made to the template based on changes recommended from usability testing. DVT Px: deep vein thrombosis prophylaxis; I&O: input and output; NYU: New York University.

The templates and hyperlinks to data visualizations were built within the sandbox testing environment of the EHR to function the same as when live in the production system. The testing protocol combined “near-live” clinical simulation of the note-writing processes with “think-aloud” protocol and task-based testing. In this pragmatic usability test, physician informaticists collected data including observational notes, notes on debriefing interviews, and real-time analysis of user-screen interactions. At the end of each testing session, participants completed the 10-question SUS. Summative analysis included a structured summary of themes from physician informaticists debriefing and session notes, average SUS scores, and response distribution by question for the 22 session participants. Themes and SUS scores were presented to the governance committee, and improvements were made to the template prior to go-live (Figure 9).

### Improvement Curriculum and Template Go-Live

Two weeks prior to go-live, all 350 internal medicine providers in the institution were assigned an electronic learning module with an introductory message from the committee chair, elucidation of guiding principles for documentation improvement, and demonstration of the new template in the electronic record. In-person roadshows were conducted in more than 15 academic conferences, quality forums, department, and division meetings across the 4 hospital campuses. The template went live on March 8, 2021. Post live, physician informaticists rounded on all 3 hospital campuses for the first week to support adoption and answer questions about the new approach to electronic documentation.

### Post Live Measurement of Note Length and Adoption Statistics

Template utilization and note length in characters were the primary outcomes measured. We used the month of November prior to provider outreach as the baseline preintervention data and the 3 months immediately following implementation in March as the postintervention data.

## Results

**Figure 10:**
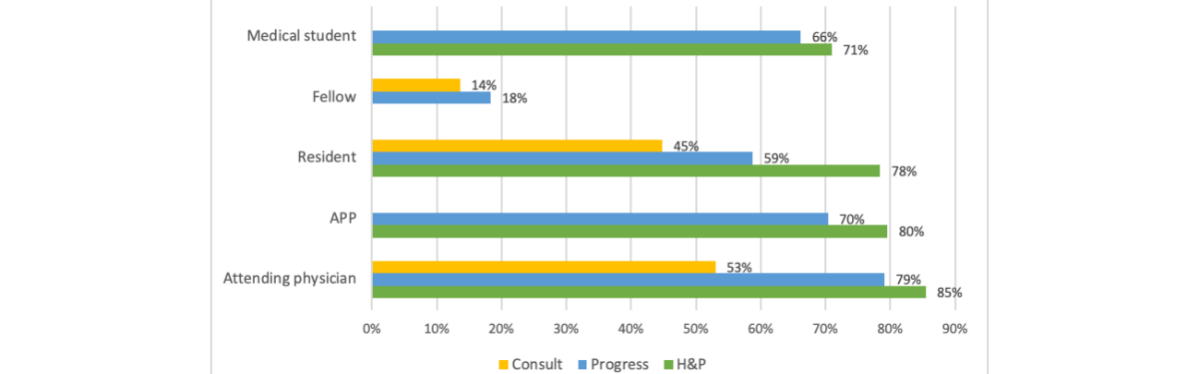
Template adoption by provider type and note type. APP: advanced practice provider; H&P: history and physical.

In usability testing among 22 medicine providers, the new progress note template averaged a usability score of 90.6 out of 100 on the SUS. A total of 77% (17/22) of providers strongly agreed that the new template was easy to use, and 64% (14/22) strongly agreed that they would like to use the template frequently. At 3 months, we observed the following template utilization by note types across 3 campuses: 75% (9048/12,142) of H&P notes, 67% (41,706/62,518) of GIM progress notes, and 34% (677/1987) of inpatient GIM consult notes were written with the new templates. We evaluated template utilization by provider type—attending physicians, advanced practice providers, medical students, residents, and fellows (Figure 10). Adoption rates for attending physicians among the different provider types were the highest with 85% (2958/3465), 79% (15,862/20,056), and 54% (547/1036) for H&P and progress notes, respectively, followed by advance practice provider H&P and progress notes at 80% (2387/3004) and 70% (5138/7352), respectively. Notes written by the fellows have the lowest adoption rates of 14% (27/199) for consult and 18% (11/60) for progress notes. H&P notes written by residents and medical students had a high adoption rate of 78% (3645/4648) and 71% (39/55), respectively, while the resident consult notes had an adoption rate of 45% (185/413). Progress notes written by residents and medical students had an adoption rate of 59% (10,821/18,430) and 66% (885/1337), respectively.

**Figure 11:**
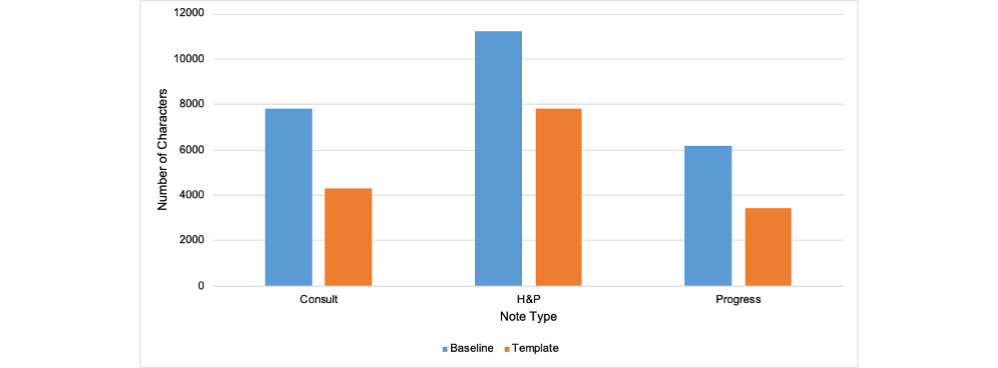
Reduction in note length of consult, H&P, and progress notes following implementation of the new templates. H&P: history and physical.

During this period of time across our institution, the length of notes written using the new templates decreased by 45% (3505/7819), 31% (3427/11,226), and 45% (2768/6191) for general medicine consults, H&P notes, and progress notes, respectively, compared to a baseline measurement period prior to interventions (Figure 11).

## Discussion

By engaging our physician leaders and gaining input from key stakeholders, we developed 11 guiding principles for general medicine documentation standards that were adopted across 3 campuses. We then incorporated these standards into our template designs along with appropriately placed clinical decision support within the SOAP structure. We further refined the templates through usability testing and feedback from clinicians representing 3 campuses. With the widespread adoption of EHRs, clinical notes have increased in length to include redundant information. This has added to the documentation burden of providers while reducing the readability of these notes. Furthermore, the direct flow of raw unprocessed data from the EHR into clinical documentation compromises the quality and usability of notes. Leveraging our EHR tools and drawing on the original SOAP note structure, we applied longstanding usability practices and note template design to make our notes readable and clinically useful again.

We used note length as a measure of readability and template adoption as a measure of template utilization. Utilization of these new templates resulted in an average reduction of 40% in the note length on the general medicine inpatient service. We had concerns that the introduction of new unfamiliar templates may not be readily accepted by clinicians who prefer their own personalized templates. However, we noted a rapid adoption of these new note templates across all 3 campuses along with high usability scores on the SUS. Attending providers and advanced practice providers had high adoption rates for the H&P and progress notes, with an average of 82% and 75%, respectively. However, we did note the lowest adoption rate by fellows with an average of 16% and a lower adoption rate of progress notes written by residents and medical students with an average of 62%. Our medical students and residents likely created note templates, which leverage their progress notes for rounding. This could result in hesitance to adopt the new templates as it would be disruptive to their rounding workflow [10]. Furthermore, the NYU General Internal Medicine department does not have fellows as part of our training program, and it was likely that other providers were incorrectly assigned as a fellow with our EHR. Our bundled intervention showed that a standardized note template that reduced the flow of extraneous information available elsewhere in the medical record was highly acceptable among most providers. Leadership buy-in with design validation is essential for effective implementation of such templates across large multicampus organizations.

This study has several limitations. This study was limited to the GIM notes. Studies would be required across other specialties to assess if a similar impact is noted in other services. In addition, consult notes had consistently lower adoption rates across all campuses. This could be attributed to a couple of different factors. Consult notes for medicine include medical clearance for surgical procedures. These consult requests were not included during the development of the consult note templates, which led to providers using their own templates to document medical optimization. We also realized that a different group of clinicians were primarily assigned to medicine consult services and were not educated about the presence of the note template, which could have led to reduced utilization of these note templates. Physician notes have several audiences including nursing, care managers, social workers, billing, coding, and other administrative staff. Feedback from such nonphysician staff members who also utilize physician notes was not included during this initial evaluation. Note length is limited as an indicator of note quality and workflow efficiency. Quality review with appropriate control for interrater reliability is necessary to further evaluate the effectiveness of the template [52]. Also, further research is needed to assess the impact of these interventions on provider efficiency [53,54]. Lastly, although copy forward was not available, the windows copy-paste function is available, and future research is needed on the impact of copy and paste on the flow of information.

In our study, using design principles, usability analysis, and stakeholder engagement, we successfully deployed standardized note templates within a hospital’s GIM service. We believe our approach can be applied to other hospital services and disseminated to other hospital systems to improve documentation standards. Further research is needed to study the impact on note quality and workflow efficiency.

## Abbreviations

CDS: clinical decision support
DIKW: Data, Information Knowledge, Wisdom
EHR: electronic health record
GIM: general internal medicine
HITECH: Health Information Technology for Economic and Clinical Health
H&P: history and physical
IPASS: illness severity, patient summary, action list, situation awareness, and contingency planning and synthesis by receiver
IRB: institutional review board
NYULH: New York University Langone Health System
SBAR: situation, background, assessment, and recommendation
SOAP: subjective, objective, assessment, and plan
SUS: System Usability Scale

## Data Availability

The data sets generated during and analyzed during the current study are available from the corresponding author on reasonable request.
